# First report of *Fasciola hepatica* seroprevalence and risk factors in European bison *(Bison bonasus)*

**DOI:** 10.2478/jvetres-2025-0020

**Published:** 2025-04-04

**Authors:** Anna Didkowska, Daniel Klich, Katarzyna Filip-Hutsch, Katarzyna Matusik, Monika Krajewska-Wędzina, Marlena Wojciechowska, Stanisław Kaczor, Wanda Olech, Krzysztof Anusz

**Affiliations:** 1 Department of Food Hygiene and Public Health Protection, Institute of Veterinary Medicine, Warsaw University of Life Sciences - SGGW, 02-776 Warsaw, Poland; 2 Department of Animal Genetics and Conservation, Warsaw University of Life Sciences - SGGW, 02-786, Warsaw, Poland; 3 Sub-Department of Veterinary Microbiology, Faculty of Veterinary Medicine, Institute of Preclinical Veterinary Sciences, University of Life Sciences, 20-033 Lublin, Poland; 4 County Veterinary Inspectorate, 38-500 Sanok, Poland

**Keywords:** antibodies, European bison, *Bison bonasus*, liver fluke, Poland

## Abstract

**Introduction:**

*Fasciola hepatica* is a trematode that infects ruminants worldwide. It is also the infectious agent of a zoonosis, fasciolosis, which is considered to be a re-emerging disease. There is no data about *F. hepatica* seroprevalence in European bison (*Bison bonasus*); however, complex population health monitoring is particularly important in protected species such as this. Addressing the need for this surveillance, the aim of this study was to assess for the first time the seroprevalence of *F. hepatica* in Polish free-living European bison populations and identify risk factors for infection.

**Material and Methods:**

Between 2020 and 2024, serum samples were collected from 119 free-ranging European bison from mountains and lowland areas. Serum samples were tested with a commercial ELISA to detect antibodies to *Fasciola hepatica*, and the data yielded were statistically analysed.

**Results:**

The study revealed *F. hepatica* seropositivity in 20/119 animals (16.8%), with higher herd seroprevalence in lowland groups, and higher individual seroprevalence in females and animals from lowland areas.

**Conclusion:**

Our study shows that a serological examination may be a useful and convenient diagnostic tool in assessing the *F. hepatica* exposure of the European bison population, especially when performing epizootic and retrospective studies.

## Introduction

*Fasciola hepatica* and *Fasciola gigantica* are the causative agents of fasciolosis, a foodborne disease with a global distribution which affects a wide range of both domestic and wild mammals (3). Human fasciolosis is considered a re-emerging disease and is favoured by climate and environmental changes (2, 27, 40). *Fasciola gigantica* is detected mainly in Africa and Asia, whereas *F. hepatica* (the liver fluke) is distributed worldwide (26). Both of those flatworms have complex life cycles, using lymnaeid snails as an intermediate host (25). The main definitive hosts of this parasite are herbivores, in which adults of *F. hepatica* localise in the biliary ducts (14). There are still not many data about *F. hepatica* prevalence in wildlife in Europe (10). There have been studies conducted on its occurrence in red deer (*Cervus elaphus*) (10), wild boar (*Sus scrofa*) (30, 39), fallow deer (*Dama dama*) (23), European rabbits (*Oryctolagus cuniculus*) (37), European hares (*Lepus europaeus*) (38) and nutria (*Myocastor coypus*) (29). It is also known that *F. hepatica* has become one of the most important parasites of European bison (*Bison bonasus*) over the years of the species’ restitution (15). The prevalence of infection reached 43 to 100% in some examined European bison populations, probably as a result of increasing contact with cattle and cross-species transmission of *F. hepatica* (6, 8).

The gold ante-mortem diagnostic standard for trematode infection involves examining faecal eggs; however, there are other diagnostic methods in frequent use, including serological ones. In them, different antigens find application for antibody detection, such as the proteases and glutathione S-transferases which the parasite uses to survive in the host body (28, 32). Complex health monitoring of the European bison population is particularly important in light of its protected species status. To date, coprological studies concerning *F. hepatica* have been conducted in European bison (5, 11). This report aims to use a serological method for the first time to assess the prevalence of *F. hepatica* in Polish free-living European bison populations and identify risk factors for infestation.

## Material and Methods

### Animals sampling

**Fig. 1.**
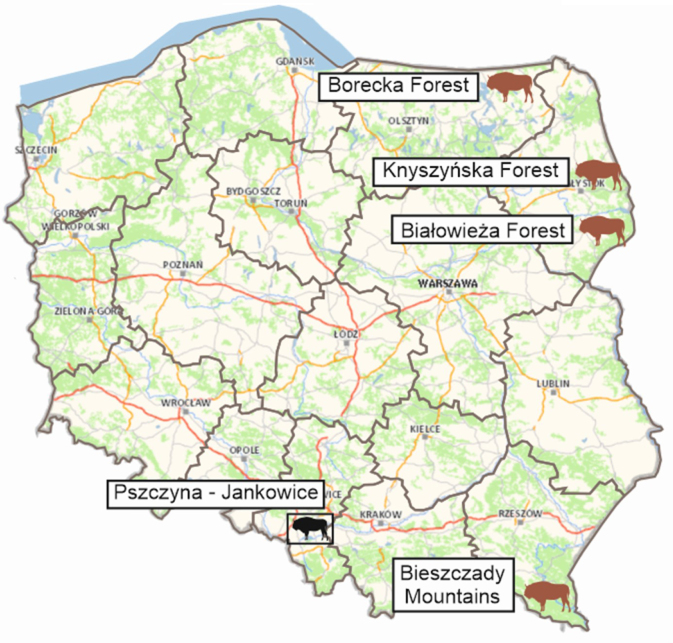
Map of Poland with marked sites of collection of material from European bison

The research was conducted in Poland as a part of the “Complex project of European bison conservation by State Forests”. Between 2020 and 2024, serum samples were collected from 119 free-ranging European bison comprising 44 females and 75 males. The animals had habitats in the Bieszczady Mountains (n = 70) or the lowland Pszczyna-Jankowice, Knyszyńska Forest, Borecka Forest or Białowieża Forest areas (n = 49) (Fig. 1). The animals ranged in age from 1 to 23 years.

No animal was immobilised or culled exclusively for this study. Samples were collected ante-mortem during planned pharmacological immobilisations (*e.g*. for putting on telemetry collars) or post-mortem following culls approved by a resolution of the General Directorate for Environmental Protection or an administrative decision of the Polish Ministry of the Environment.

Blood was collected from the jugular vein into 6-mL serum tubes with a clot activator. Tubes with blood were transported to the laboratory under refrigeration. After centrifugation at 1500 × *g* for 10 min, the obtained serum samples were stored at –20°C until further analysis.

### Diagnostic analysis

After being defrosted and brought to room temperature, the serum samples were tested with an indirect Monoscreen Ab *Fasciola hepatica* ELISA (Bio-X Diagnostics, Rochefort, Belgium). The test was performed in accordance with the manufacturer’s manual. Briefly, the principle of the test is based on using microtitration plates sensitised by a monoclonal antibody specific to one protein of *F. hepatica*.

The plate’s odd-numbered columns contain specific proteins, and its even-numbered columns contain only monoclonal antibodies to differentiate specific anti-*F. hepatica* antibodies from non-specific ones. The optical density of each sample was read using an EPOCH spectrophotometer (BioTek Instruments, Winooski, VT, USA) at a wavelength of 450 nm and calculated in accordance with instructions. The degree of positivity was based on antibody titre and was interpreted as follows: 0 indicated no *F. hepatica* infestation, +/– was noted as a dubious outcome, + showed low-grade infestation, ++ was a moderate infestation result and +++ was a sign of heavy infestation. If available, post-mortem protocols were consulted to check whether a liver fluke was detected.

### Statistical analysis

Using infestation degree as a response variable, the effects of sex and age of animals and location (mountains/lowlands) on *F. hepatica* infestation were analysed. For this comparison, a generalised linear model with Poisson distribution was applied. All model variants including the null model were constructed to find the highest-ranked model with the lowest Akaike information criterion value based on Burnham and Anderson (4). Animals with liver flukes discovered in post-mortem examination were compared to those without liver fluke with a U Mann–Whitney test (Z test) with regard to the degree of *F. hepatica* infestation. All statistical analyses were performed with IBM SPSS Statistics 29.0 (Armonk, NY, USA).

## Results

**Fig. 2.**
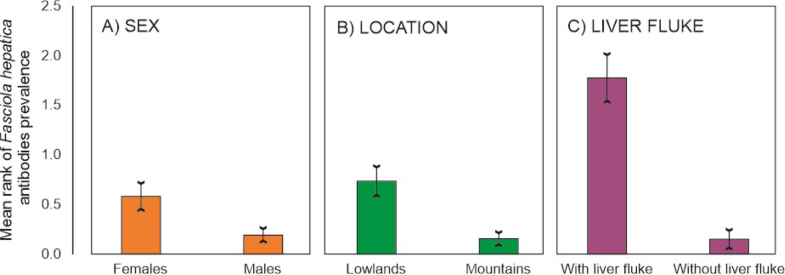
Mean (±standard error) seropositivity of *Fasciola hepatica* in European bison with regard to sex of animals (A), site location (B) and presence of liver fluke (C). The graph presents marginal means from the generalised linear model in A and B, and average values in C

The study revealed *F. hepatica* seropositivity in 20/119 animals (16.8%). Statistical analysis showed a significant effect of sex and location on *F. hepatica* seropositivity in European bison (Table 1 and Fig. 2). Females were infected to a higher degree than males (beta coefficient (B) = 1.090, P-value < 0.001). Animals in the lowlands were more likely to be seropositive than animals from the Bieszczady Mountains (B = 1.544, P-value < 0.001). The age of the animals did not influence the degree of the seropositivity, and the variable was excluded from the model (Table 1).

**Table 1.** Effect of sex and site on Fasciola hepatica seropositivity in European bison. The age variable was excluded in the model selection procedure

	B	SE	Lower CI	Upper CI	Wald chi^2^	P-value
Intercept	–2.392	0.3611	–3.099	–1.684	43.872	<0.001
Female	1.090	0.3087	0.485	1.695	12.462	<0.001
Male	0*					
Lowland location	1.544	0.3433	0.872	2.217	20.243	<0.001
Mountain location	0*					

1 B – beta coefficient; SE – standard error; CI-confidence interval;

* – redundant category

Animals with liver flukes discovered in post-mortem examination presented a significantly higher degree of *F. hepatica* infestation than animals without them (Z = 5.393, P-value < 0.001).

## Discussion

This report is the first determination of the occurrence of *Fasciola hepatica* antibodies in the European bison population. Our report has shown that 16.8% of tested animals were seropositive, which is a close result to that of the latest studies based on coprological examination giving the prevalence of *F. hepatica* eggs in faecal samples at 13.1% (11). We have also shown the influence of the presence of *F. hepatica* on the occurrence of antibodies, which indicates the high value of the serological test for such monitoring in the European bison. Taking into consideration that *F. hepatica* is widespread in cattle in Poland (18, 35) and contacts between cattle and European bison may well take place (15, 16), it seems to be highly important to control this pathogen. Previous reports have shown that *F. hepatica* is noted in the Białowieża Forest where there are no cattle; therefore, the terrain is also a very important component in determining the risk of fasciolosis in European bison (20, 21, 22).

Serological monitoring of *F. hepatica* in livestock herds is gaining popularity as a diagnostic tool (7, 13). It has some advantages over standard faecal egg identification, one of which is the ability to identify infections much earlier (at 4–5 weeks after acquisition) in both animals and humans (12, 24, 31). In wildlife, faecal egg counting is difficult because the processing of fresh material is required before its desiccation or freezing, which can damage eggs. In this case, coproantigen ELISA or serological studies can be more practical (10).

Our study reveals that infestation is higher in lowland areas than in mountainous ones. This is not surprising, given that that lowland areas are covered by wetland to a higher degree and that there is a higher risk there of bison encountering snails, which are *F. hepatica* intermediate hosts (1). The occurrence of snails in the Bieszczady Mountains is limited to lower pastures, not inhabited by European bison during the vegetation season. Therefore, trematodes have only rarely been detected in European bison in the Bieszczady Mountains (5) and the carriage rate in this particular population is low, which was confirmed in our study. This relationship has been also observed in ruminants in other mountain ranges (33, 34, 38).

A higher level of seropositivity in females may be the result of the species behaviour of European bison, as females live in groups, while males mainly do as single free-ranging individuals (19, 36). Furthermore, the immunosuppressive effect of pregnancy and lactation might facilitate infection and explain the observed pattern (22). Sex-biased parasitism has also been commonly observed in European bison by other authors (9, 17).

Our study has some limitations. The best solution to assess *F. hepatica* prevalence in European bison would be to use several methods simultaneously. In the future, it would be worth conducting not only antibody ELISAs but also coproantigen ELISAs on European bison samples as an *F. hepatica* diagnostic tool. Another limitation of this study is that the results could not be compared to the macroscopic assessment because post-mortem protocols were not available for all animals, or because serum was collected ante-mortem.

## Conclusion

Our results revealed that 16.8% of tested European bison were *F. hepatica*-seropositive, with higher prevalence in females and animals living in lowlands. Animals with liver flukes found in post-mortem examination presented a significantly higher degree of *F. hepatica* infestation. Our study shows that a serological examination can be a useful and convenient diagnostic tool in assessing the *F. hepatica* exposure of the European bison population.
